# Religion and Health: exploration of attitudes and health perceptions of faith healing users in urban Ghana

**DOI:** 10.1186/s12889-018-6277-9

**Published:** 2018-12-10

**Authors:** Prince Peprah, Razak M. Gyasi Mohammed, Prince Osei-Wusu Adjei, Williams Agyemang-Duah, Emmanuel Mawuli Abalo, Josephine Nii Amon Kotei

**Affiliations:** 1 Department of Geography and Rural Development, Kwame Nkrumah University of Science and Technology, Kumasi, Ghana; 2 African Population and Health Research Center, Manga Close, Off-Kirawa Road, P.O. Box 10787-00100, Nairobi, Kenya

**Keywords:** Faith healing, Health perception, Attitudes, Health seeking behaviour, Well-being, Kumasi Metropolis, Ghana

## Abstract

**Background:**

The main aim of the study was to explore the attitudes and health perceptions of faith healing users in Kumasi Metropolis, Ghana. This has become necessary because faith healing practice is an important area but remains neglected in the health care literature. In an age when biowestern medicine is touted as the cure for most diseases, understanding how and why individuals seek alternative treatment, specifically faith healing modalities may help to develop more effective health care interventions.

**Methods:**

We employed exploratory study design of purely qualitative research approach involving 40 conveniently selected participants from four different purposively selected faith healing centres to get a maximum variation of experiences and opinions on the time of consultation, perceived effectiveness and challenges of faith healing practices in Ghana. In-depth interviews were conducted from 10th June to 30th July, 2017. Data were thematically analysed and presented based on the a posteriori inductive reduction approach.

**Results:**

The main findings were that faith healers served as the first port of call for disease curing and prevention for most users. Consumers of faith healing perceived their health status to be good due to the perceived effectiveness of faith healing for curing of health problems. However, users faced challenges such as stigmatisation and victimisation in seeking health care.

**Conclusion:**

This study has provided the first baseline evidence in this important area of inquiry that has been neglected in the scholarly discourse in Ghana. By implication, users’ positive attitudes and perceptions toward faith healing call for integration policies that allow formal medical services to have open idea to faith healing practices in Ghana.

## Background

The practices of faith healing in the diagnosis, prevention and treatment of a plethora of health issues is pre-historic and dates back into antiquity in many countries [1–3]. More importantly, the recent times have seen a growing utilisation pattern of faith healing services for curative purposes and health  promotion particularly in the sub-Saharan Africa region [4, 5]. Faith healers are usually professed Christians who belong to either mission or African independent churches, or traditionalists in form of animists who mostly heal through prayer, laying hands on patients, providing holy water and medicinal herbs [6]. Faith healers believe that their healing power comes from God through ecstatic states and trance-contact with a Christian Holy Spirit and/or ancestral spirit [7].

The increasing utilization of faith healing services could partly be attributed to the rising awareness in its complete healing process. In treating patients, faith healers view health and diseases through the integration of mind, body and spirit largely within the context of family and community [8]. This implies that the healers deal with the complete person aside providing treatment for physical, psychological, spiritual and social symptoms. The approach of faith healing goes beyond the World Health Organisation's (WHO) definition of helath as “a state of complete physical, mental and social wellbeing and not merely absent of disease or infirmity” [9].

Notably, a significant number (66.7%) of patients sought the services of faith healers because of the fact that faith healing is often readily available to potential consumers  [4]. However, regarding other kinds of treatment such as bio-western medicine, patients often travel longer distances and encounter longer waiting times to access the needed health care  [4]. Whereas faith healing services are affordable, the decisions to use such services are motivated by trust, availability and accessibility, recommendations from relevant other and believe in the supernatural causation of illness [4, 10]. Evidence also shows that neglecting spiritual needs results in less favourable outcomes for patients such as reduced quality of life, dissatisfaction with care and increased costs at the end of life [11, 12].

However, investigating the effectiveness of faith healing using scientific inquiry is controversial while there is no or limited evidence of therapeutic effectiveness of faith healing modalities such as prayer [13, 14]. Nevertheless, it is evident that a significant proportion of patients resort to faith healing as a first choice in different cultures and also believe in its potency [10, 15].

Patients all over the world seek the services of faith healers for all kinds of ailments including social and psychological issues. However, some faith healers specialise in dealing with specific health problems and in emergency cases, refer patients to formal health care providers for treatment [16]. Aside from the reassurances of faith, concerns have been raised regarding the individuals who practice faith-healing; patients are often exposed to a combination of herbs, remedies, chemicals, holy water, and other assumed treatment substances which are often harmful leading to physical problems [17, 18].

Thus, there is a burgeoning research interest and recognition to explore, the effectiveness of faith healing by researchers and health care providers. Even though, there has been growing research on the relationship between faith and health globally, there is paucity of research on this subject in the Ghanaian context especially regarding attitudes and health perception of faith healing users which could inhibit the attainment of United Nations Sustainable Development Goal 3 of ensuring health and wellbeing for all at all ages by 2030. Moreover, the proliferation of faith healing churches and centres in Ghana and the general interpretation of diseases and illness causation within a religious context [19], justify the need for this study. Furthermore, the limited knowledge about the attitudes and health perceptions of faith healing tends to limit the policy adoption and implementation in regulation to  faith healing practices. It is believed that the understanding of the attitudes and health perceptions of faith healing among users' is critical  following the escalating use of faith healing modality in Ghana. It is, therefore, apt and timely enough to conduct this study to provide baseline evidence in this important but less explored area of inquiry in Ghana. This might also serve as a useful reference for other settings where the use of faith as a healing modality is predominant. This current study, therefore, aims at exploring the attitudes and health perceptions of faith healing users among adult population in Kumasi Metropolis, Ghana.

## Methods

### Study design and setting

This exploratory qualitative study was conducted in four purposively selected faith healing centres within Kumasi Metropolis, Ghana. Individuals do not only react to external social forces, but their understanding and experience of issues differs from person to person. For a qualitative study which sought to understand the experiences, opinions and attitudes of people in their own context, the interpretivist paradigm and subjectivist epistemology were adopted [20]. In line with this paradigm, the respondents were given the freedom to interpret, describe and explain their opinions and feelings about the problem under study in their own context. With this approach, the original feelings, experiences and belief systems of participants were valued. These perspectives ensured adequate discourse between the researchers and the interviewees to generate a meaningful collaborative effect [21]. This research orientation was appropriate because it helped to avoid rigid structural paradigms, such as those in positivist research, and adopt more personal/ flexible research structures, which are receptive to capturing meanings in human interaction and make sense of what is perceived as reality. This approach creates room for both the interviewer and the interviewee to establish a good rapport which stimulates their discussion to generate new ideas to enhance the study. Kumasi Metropolis which falls under the jurisdiction of Ashanti Region of Ghana was selected for this study as a result of two main reasons. One, it is noted as a multi-ethnic geographical area with varied socio-demographic and cultural characteristics of the population. It also attracts migrants from different origins and socio-cultural settings because of its dominant socio-economic potentials. This has resulted in high population density with resultant effect of high morbidity burden among vulnerable groups [22]. It is also classified as a semi-deciduous forest landscape with abundant medicinal plant for the treatment of diseases. Second, aside the relatively numerous healthcare facilities and professionals, spiritual and faith healing modalities are currently widespread. These provide a diversity of healing choices at the disposal of the community members for their health care requirements in the Metropolis.

### The sample and sampling procedure

In this study, we purposively and conveniently selected four faith healing centres with 40 participants (faith healers and users) to get a maximum variation of experiences and opinions on the time of consult, perceived effectiveness and challenges of faith healing in Ghana. Although we recognise that randomize sampling would have been a better method to remove bias, especially in deciding which faith healers to be included in the study, the lack of reliable adequate baseline data on this population made the use of such a technique largely unviable. The use of purposive sampling offered the required flexibility to focus on participants who met the study criteria. Four main criteria were used in the selection process: the centre being a faith healing church; being a non-church faith healing centre; being in existence for more than ten years and; being popular to the general population. Based on these criteria, the four centres were recruited in the study comprising two Christian faith healing centres and two Islamic faith and Traditional faith healing centres respectively. The selection procedure, to a larger extent, was an arbitrary one, which did not take into account any of such parameters as the size of the target and the accessible population of the study areas, following [23]. Two aggregated sets of participants were included in this study, these were faith healing users (*n* = 36), and healers (*n* = 4). The faith healing users encompassed lay individuals who had used or were using prayer, holy water, ointment, medicinal plants and other forms of traditional medicines for self-health care, self-treatment prescribed by a faith healer. In this study, a faith healer was tailored as a professed Christian, Islamist or Traditional spiritualist who heals by application of faith mostly through prayer, fasting, holy water, ointments and medicinal plants.

Other specific inclusion criteria were: age 18 years or older as people contribute to national decision and policy discussion at this age, at the premises as of the time of the interview and self-reported knowledge regarding some levels of time of consult, effectiveness and challenges of faith healing and the knowledge of diagnosis and treatment/management of health issues.

### Data generation tool and procedure

An in-depth interview was the tool for data collection. In-depth interviews were used to generate data and an interview-guide developed to ensure that similar themes and questions were covered in each discussion and interview. The interviews were systematically conducted between a participant and an interviewer at the place where the participant was recruited, mainly at the faith healing centres. The interviews were conducted in “Twi” which is the local dialect of the study area by the researchers themselves in an enclosed space that was free from interference by any third party. With the consent of the participants, interviews were audio-recorded while field notes were also taken. The opening question asked users to provide details of their experience about faith healing practice. Users were also asked to share their views on the perceived effectiveness of specific faith healing modality they used and to give examples of situations in which they felt faith healing had contributed to disease treatment. The healers were first asked to provide a general description of their services. They were also asked to offer explanations for the recent upsurge use of their services and to provide specific instances where they have used faith to heal or cure a particular disease. Data credibility and rigor was enhanced through prolonged engagement with participants; each interview lasted approximately 60 min  and was supported by field observations and the participants’ checks and validation. Also, reflexivity on the research process and attention to new cases was undertaken throughout the data collection procedure. Our study participants were assigned pseudonyms.

### Data analysis

Audio records were transcribed into both “Twi” dialect and English language of which those with “Twi” were later translated into English by all the authors individually, and cross checked with the audio records and handwritten field notes to ensure validity, reliability and quality control. The study employed the a posteriori inductive reduction approach to develop consistent themes [24]. The data was subjected to thematic and content analysis where coding and analysis is used to identify themes and subthemes [25]. As a result, we classified and organized data according to key themes, concepts and emergent categories. Themes were compared with the responses to identify common trends, similarities and contrasts. This helped the authors to do clear and detailed analysis [26]. It further provided an enabling environment where the researchers were able to establish trends in the data collected which then facilitated the analysis and reporting of results [25]. In this case, the interviewees were queried to confirm the correctness of both the main themes and sub-themes generated to ensure precision. Therefore, the results were presented based on seven major themes with the aid of direct quotations.

## Results

The findings of the study reflect the perspectives of the perspectives of the participants on attitudes towards faith healers, time of consult of faith healers, perceived effectiveness of faith healing and challenges associated with faith healing in the Ghanaian context. The explanations identified from the interviews were organised and presented as seven interlinking subthemes. Tentative themes developed from the analytical process were: Users’ knowledge and sources of awareness of faith healing; time of consult of faith healers; users’ attitudes towards faith healers; health perception of faith healing users; perceived effectiveness of faith healing; perceived safety of faith healing; and challenges of faith healing use.

### Background characteristics of the participants

Overall, 14 males (3 healers and 11 users) and 26 females (1 healer and 25 users) took part in the study. Out of the 36 users, 14 were users of Christian faith healing services, 9 were users of Traditional faith healing services with 8 using Islamic faith healing services. Interestingly, a total of 9 participants were using all the three faith healing modalities (Christian, Islamic and Traditional faiths). Out of the 9 who were using all the three faiths, 4 were Christians, 2 were Muslims with the remaining 3 belonging to the Traditional faith. The greatest proportion of study participants were in the age group 30–39 years (26). Most participants were unemployed (27) and those employed were dealing in informal economic activities. With respect to the healers, 2 were Christian faith healers with the remaining 2 being Traditional and Islamic faith healers respectively. Table 1 presents detailed characteristics of the study sample.

**Table 1:** Sample Characteristics and Type of Faith Healing Served

	*N* (40)
Sample Characteristics	
Type of respondents	
User	36
Healer	4
Gender (Users)	
Male	14
Female	26
Gender (Healers)	
Male	3
Female	1
Age (Users)	
20–29	5
30–39	26
40–49	4
50 and above	5
Age (Healers)	
30–39	1
40–49	2
50 and above	1
Level of Education (Users)	
None	3
Basic	5
Secondary/Vocational	17
Tertiary	11
Level of Education (Healers)	
Secondary	2
Tertiary	2
Employment Status (Users)	
Employed	13
Unemployed	27
Employment Status (Healers)	
Employed	4
Occupational Type (Users)	
Formal	9
Informal	31
Occupational Type (Healers)	
Informal	4
Religion (Users)	
Christianity	33
Islam	1
Africa Traditional Religion	6
Religion (Healers)	
Christians	2
Muslims	1
Traditionalists	1
Type of Faith Healing Service	
Christian Faith healer	14
Traditional Faith healer	9
Islamic Faith healer	8
All the three	9

### Users’ knowledge and sources of awareness of faith healing

All the users have a great deal of knowledge about faith healing. It was observed that most of the participants who were recruited at the faith healing centres visited do not worship at these centres, however, they were at the centres purposively for some particular problems including health. These participants were true members of different worship groups. The users of these centres mostly got to know of them through friends, relatives and the mass media. The recommendations made by friends and the constant advertisements by the mass media spread information about the faith healers who were not previously known to the users. The effectiveness of faith healing made people recommend to others who are suffering from a similar health problem:

*I got to know this place through a friend. She recommended this place because my friend was healed of the same disease I am suffering from.*

Another user also noted that:

*I once watched this healer on the television and I was pushed to come for treatment for my menstrual pains and I have been healed.*

The healers also confirmed that their clients got to know them through similar ways mentioned by the users:

*I think people got to know us through recommendations of previously healed people who came here. Most at times people who have been healed go out to recommend us to their friends and relatives who are also suffering from similar conditions.*

With the knowledge and information, they have acquired about faith healing, participants attempted to describe what faith healing and healers are respectively. On the part of the Christian faith users, faith healing is the process of using ones’ faith to cure, manage and prevent diseases through prayers, fasting, application of holy water and ointments prescribed by a Christian leader whilst a faith healer was someone who uses prayers and other forms of Christian interventions through God to heal diseases. One Christian faith user expressed that:

*I think faith healing is using your faith through prayers, fasting and other materials such as holy water, oil and ointment basically upon the instructions of your Christian leader to cure or prevent diseases. Concerning faith healers, I see them to be the leaders who pray, guide and direct us whenever we are sick. So, I see the leader of this prayer camp as a faith healer because he uses prayer and fasting to heal many diseases.*

To the Traditional faith users, faith healing was conceived as the pouring of libation to the ancestors for healing of long standing health problem(s). Though they do not physically see the ancestors who are believed to be the healers unlike Christian faith users who mostly have direct contact with the healers, traditional faith healing users believe that the ancestors hear, listen and act upon their requests accordingly, after performing the needed rites:

*To me, faith healing is believing in our ancestors that they can heal, after pouring of libation and performing other rites. Though we do not see them we believe they can heal  all manner of diseases, especially long-standing ones.*

The Islam faith users viewed faith healing in two ways: the application of what the Holy Quran says about healing and; the consultation of Islamic leader [Malam] for instructions and prescriptions as to how to go about dealing with a health problem and described faith healers as Muslims leaders who assist people in dealing with health challenges:

*For us Muslims, faith healing is all about doing what the Holy Quran tells us to do when we are sick or faced with a health challenge. Is all about believing that Allah heals. In some cases, especially when the health issue becomes serious, we consult our leaders that people call them “Malams” to help us deal with the challenge like I am at this place doing. I see both us faith healing.*

The healers see themselves as individuals who help assist people to deal both economic and social issues including health through the application of faith:

*We are individuals who belong to a particular faith, be it Christian, Islam or Traditional to deal with many life challenges including health. For example, I am a Christian leader, so I heal people through the application of the Christian faith. The same thing applies to both Islamic and Traditional leaders.*

### Time of consult of faith healers

Faith healers served as the first port of call for disease curing and prevention. On the curing, we observed several things that go through the process from the centres. Specifically, at the Christian centres, we observed that, aside the reassurance of faith through prayers and fasting, patients were offered ‘holy’ oil and water by the leaders who were themselves healers to be applied at the parts of their body they were suffering from. The application of these water, oil and ointment were not only to cure the present diseases but also to prevent future diseases from coming. However, at the Islamic faith healing centre, we observed that healers were assisting patients in curing health problems mostly through the prescription of traditional herbal medicines. Here, prayer and fasting were not mostly used by the healers. Also, the application of ‘holy’ oil and ointment were uncommon unlike the Christian faith healing centres. The users believed that these herbs prescribed by the healer can heal them. This event was similarly observed at the Traditional faith healing centre. Here, the users were only consulting Traditionalist and Herbalist for herbal medicines, aside pouring of libation. The consultation of these healers was based on the strong belief they have in the ancestors.

The main reason accounted for why users first consult faith healers before medical care was the notion by most of the participants that “Jesus is the first point of contact in everything”. This notion was, however, common among the Christians who participated in the study. As a results, the Christian faith users explained that consulting a faith healer who is “a man of God” on health issues in the first place is appropriate and required by any person who “truly” professed a Christian faith:

*Mostly, I first consult my pastor on my health issues before taking it to the hospital upon his recommendation. I do so for the purpose of ascertaining the origin of the sickness, it be natural or spiritually-motivated. At times some diseases are not natural but originate from various external sources such as witchcraft, ancestors and curse which need to be tackled through spiritual means. Also, he [healer] is the leader who is leading us in diverse ways, including both physical and spiritual, therefore consulting faith first is the right thing to do. Also, I have faith that I will be healed by God through him. If he says I should seek formal healthcare then I go, if he says I would be healed through faith, I believe so.*

On the part of the Islamic and Traditional faiths users, the decision to consult faith firstly before seeking treatment at the hospital was premised two main factors: respect for their leaders, who are mostly the healers and the belief that faith heals. The users explained that bypassing their leaders to seek medical care shows disrespect to them, hence their decision to consult them for treatment in the first instance:

*I think in everything you need to consult your leader first before any further action. I consult my spiritual leader first because I respect and believe in him. When I do not consult him and go ahead to seek medical care, is a sign of disrespect.*

It emerged from the interview that the healers encourage users to consult them first before seeking medical care upon their recommendations and instructions. This was confirmed by one healer:

*I always advice my members to consult God first in any situation including health issues. This is because faith really heals and heals faster than the scientific process of healing. I can tell you emphatically that those who spend time seeking healthcare from the formal healthcare system before coming here mostly do not receive treatment and die as a result. So, I think consulting your faith on health issues first before other alternatives is a right thing to do.*

Basically due to this, we discovered that healers are performing the role of formal healthcare professionals in health delivery process. For example, it emerged from the interviews that, as most users first visit faith healers for various health issues, some healers administer cross-referrals of patients. It was further observed that the lethargic attitude of healers to administer referrals centered on both theoretical and philosophical underpinnings of the faith healing modality system. For instance, both healers and users shared the view that most diseases are strictly spiritual, which is difficult to explain and cure in scientific terms. Also, the healers expressed that seeking health care from formal healthcare delivery first before resorting to ones’ faith delays his or her healing process, all because faith heals faster than scientific applications:

*Occasionally, I tell people who come to seek treatment to go to the hospital. I do this after I have realised that the sickness is not spiritually-motivated.*

Another healer also noted that:

*I sometimes refer patients who come here to seek medical treatment from the hospital. This is because, I acknowledge the fact that not all diseases could be cured by faith, some needs medical attention.*

### Users’ attitudes towards faith healers

The study participants regarded faith healers as their “spiritual or God fathers” who intercede for them concerning a number of issues including health. Some of the participants mentioned that faith healers serve as their source of spiritual knowledge and interpretation. It was recognised that faith healers offer to users a few services including prayers for curing and preventing of diseases that emanate from both physical and spiritual realms. With this, faith healers were not only consulted on health issues but personal issues as well. Participants maintained that faith healers are everything to them and cannot do without their services. The study found among other things that faith healers have the utmost respect of the users and vice versa:

*Faith healers are everything to my family. They are our source of healing. I really respect them. I see faith healers as spiritual fathers who see all diseases that are about to affect the body and prevent them from coming. To me, I am nothing without my church leader [healer].*

Another Christian faith user also emphasized that:

*My religious belief teaches me to have more respect for my leader who is leading me to heaven than any other person. This leader is my healer, preacher and protector both physically and spiritually.*

Interestingly, the study participants compared their attitudes toward faith healers and professional medical practitioners. Most of the participants expressed good attitudes toward faith healers more than conventional medicine practitioners. Various reasons were reported by the study participants to account for the users’ good attitude towards faith healers. The holistic treatment approach of faith healing was the main reason for seeking health service from faith healers. Furthermore, factors such as good interpersonal relationships and religious and cultural acceptance were broadly highlighted by the study participants to account for the good attitudes toward faith healers:

*Faith healers really command respect than medical doctors. We have enough respect for them because they cure both physical and spiritual illness. They also have time to listen to our complaints, devote time to teach us and explain spiritual events to us. To me, they are our source of good health so we regard them as such.*

### Health perception of faith healing users

Most of the study participants perceived their health status to be very good and attributed it to the use of faith healing. Most of the participants perceived their health status as poor before the use of faith healing. However, most of the participants had witnessed significant improvement in their health status since they started using faith healing services for curing and preventing disease. In addition, participants who were once admitted at formal healthcare facilities before visiting faith healers considered their recovery process as faster compared to when they were at the formal health care facilities. As a result, some of the participants who were receiving treatment at the visited centres described their health status as good compared to before using faith healing:

*I frequently complained of headache and stomach ache but since I started using faith healing, I hardly fall sick. Before using the herbs prescribed by this faith healer, [the healer at one centre visited] I was buying orthodox medicines prescribed by doctors to cure “common” headache and stomach ache but those medicines could not cure it until I found faith healing. Now I can say my health status is really good compared to before using faith healing.*

One Christian faith user similarly noted that:

*I can say my health status is very good. I must attest to the fact that my health status was not good when I was using orthodox medicines for disease treatment. So with faith through prayers, fasting, anointing oil prescribed by my pastor, my health status has been very good.*

The issue of whole person treatment were echoed by the participants to explain the perceived good health status. Participants elaborated that faith healing takes into account the complete human being and deals with it holistically. Specifically, the Christian faith users mentioned that due to prayer and fasting, diseases that were supposed to be emanated from the spiritual realms were, therefore, prevented, hence promoting their health and welfare. On the part of the Islamic and Traditional faith users, the traditional herbs and the pouring of libation prevent regular occurrence of diseases. In all, participants maintained that faith healing had been an important healing modality for them because of how it had improved their health status.

*I spent more than two months at a certain hospital in this metropolis seeking treatment. Initially, the doctors told me I was suffering from stomach ulcer and later informed me that I had contracted typhoid fever also. I bought and used a number of drugs but to no avail. A friend recommended this healing centre to me. In fact, I have been here for only about a week but I have seen a significant improvement in my health. Here, the healer only prays with us and apply holy water, ointments. I see this form of treatment to be holistic. Because, the prayer and the ointment do not heal one sickness but many sickness.*

### Perceived effectiveness of faith healing

All the users perceived the effectiveness of faith healing modality as very good for curing diverse forms of health problems. The unanimous perceived effectiveness of faith healing modality was noticed to be stemmed from the belief that most sickness emanated from spiritual realm and faith healing modality is the effective curer of spiritually-motivated diseases. The participants were of the belief that certain diseases have strong spiritual connections and underpinnings, thus can only be treated and reversed effectively through spiritual means, particularly by prayer and fasting:

*I am a living testimony. This is because I came to this place doubting the effectiveness of prayer for healing. I had a broken leg while playing football. I have been to different formal healthcare facilities but yielded no positive improvement for about five years. One of my relatives recommended this prayer camp to me but I was initially doubtful. When I eventually agreed and was sent to this place for only almost three weeks, through prayers and other ointments, I am able to walk. I can emphatically say that faith healing [prayer] is very potent and effective.*

### Common disease types that participants seek for help

Specifically, some cases of diseases which have been healed through faith were broadly highlighted including mental problems, malaria and typhoid fever, sexually transmitted infections, infertility, chicken pox, menstrual problems, sexual weakness, low sperm count, piles, cold, influenza, cough, hernia, intestinal problems and bone fracture:

*To me, spirituality is the cause of almost all diseases and must be healed through spiritual means. My daughter was seriously suffering from infertility. She was married for about six years without a child. The interesting part was that, doctors found no problem with her and the husband yet, they could not give birth. I took both my daughter and her husband to this place and was told that her womb was sealed by a family member who is jealous of her marriage. We prayed, fasted and applied certain oil and instructions given by the healer, now they have two kids.*

The healers also agreed with users on the perceived effectiveness of using faith for healing. To the healers, faith is the master healer hence, mentioned some specific cases of illness that have been healed by them through the application faith:

*Mostly, many people do not believe in the healing power of faith until they have their own experiences. I must tell you that I have healed so many people with different health issues including psychiatric issues through faith. All people you are seeing here came with severe health problems but now have their problems almost gone or improved.*

One Traditional faith healer also explained that:

*My brother….I must tell you that faith really heals, in fact, no drug in this world heals more than faith. I am the healer and knows a number of people with diverse health issues that I have been able to cure and prevented through faith. We have so many ways that diseases come to affect the human body. Either physical or spiritual and at times combination of both. For the physical one, orthodox can cure but for the spiritual diseases one needs to tackle them through his or her faith in the ancestors that they can heal.*

### Perceived safety of faith healing

Aside the perceived effectiveness of faith healing, interviewees discussed the safety of faith healing modality. It was observed that participants perceived most aspects of faith healing such as prayer, fasting and herbs to be devoid of side effects unlike the orthodox medicines. In view of this, some section of the participants expressed that part of their reasons for the use of faith healing was due to the fact that it is perceived to be more natural:

*Prayers are free from chemicals which threaten health unlike manufactured drugs. Also, at times, herbs that are given to us by the healers are mostly natural plants free from chemicals that can cause damage to our health. Personally, I see faith healing as safe and effective.*

Another user also mentioned that:

*Faith healing is very safe and effective. I do not have to take drugs that have serious side effects. I just pray and believe that I would be healed.*

### Challenges of faith healing use

Specific evidences were given by participants regarding the challenges of faith healing use. One major concern raised by the participants was related to the stigmatisation and victimisation. However, these concerns were reported commonly among the users of Traditional faith healers service. Users of traditional faith healers service explained that society mostly stigmatised and victimised them for not using formal healthcare services but rather opted for Traditional faith healing modality:

*Many people in my neighbourhood do not want to come close to me because I visit spiritualists on health issues. They see me as an unbeliever, sinner and devil. It is about time people understand people’s choice of healing.*

Additionally, issue of lack of respect from community members was also broadly mentioned by the Traditional and Islamic faith users. They expressed that many people within their communities do not respect their choice of healing modality and looked down on them as many people perceive the use of ancestral homes and Islamic faith healers [Malams] as a sin.

*People in my community look at me with a different eye because I visit ancestral homes for health treatment. They look at me as a sinner and do not even respect me. I think this is a challenge.*

Similar lamentation was shared by another participant:

These challenges were confirmed by the healers:

*I mostly receive complains of stigmatisation and lack of respect from many people who come here for health treatment. To me, it is a serious challenge and needs to be addressed. Going forward, I think faith healing modality should be integrated into the mainstream healthcare system for coexistence.*

## Discussion

This study examined the attitudes and health perceptions of faith healing users in urban Ghana. The demand for faith healing services in both developed and developing countries continues to escalate. In this case, various studies have been conducted on some aspects of faith healing modalities [4, 5, 27–30]. Thus, to contribute to this debate, the present study has detailed the spectrum of attitudes and health perception of faith healing users in Kumasi Metropolis, Ghana. Also, sources of faith healing information, effectiveness, challenges and patients’ time of consult have been explored in this study.

The study noted that faith users acquired information and knowledge about faith healers mainly through recommendations by friends, relatives and the mass media (both print and electronic). It was evident that those who had been healed through faith most often recommend to friends or relatives suffering from similar conditions. This finding apparently concurs with other previous studies that friends, the mass media and family members are the sources of information on the use of faith healing services [22, 31].

It was noted that the faith healers owned a number of the media outlets with the purpose of projecting events of healing and other cases at their various centres. This finding implies that information and awareness of faith healing modality is more likely to spread across the population in the region considering the increasing spread of these media outlets in Ghana. Thus, more people are also likely to get to know about faith healers and thereby use their services for healthcare.

Significantly, the present study revealed that faith healers served as the first port of call for a considerable number of the participants for primary healthcare needs despite the current advances of conventional treatments. It was observed that most of the participants suffering from minor and major illness sought help from faith healing before probably seeking medical treatment, usually upon the instructions and recommendations of the healers. This finding supports results from other studies that faith healers remained the first port of contact for most patients in dealing with their health problems. This is attributed partly to the belief systems in most societies and how these beliefs influence patients’ perception regarding the causes of diseases [4, 10, 15, 29, 32]. Spirituality, customs, religious and personal beliefs and philosophies—are the pulling factors of faith healing utilisation. The study observed that, participants believed that explanations on the causes of diseases must be known first before application of medicine. Henceforth, knowledge about the causes of diseases helps proffer appropriate solutions. Since patients consult faith healers first before attending to medical treatment, some healers administer cross-referrals of patients. However, such referrals seldom occurred when there is absence of emergency cases. It was further observed that the lethargic attitude of healers to administer referrals centered on both theoretical and philosophical underpinnings of the faith healing modality system. For instance, both healers and users shared the view that most diseases are strictly spiritual, which is difficult to explain and cure in scientific terms whiles faith heals faster than scientific applications. This observation is certainly in line with Campion and Bhugra [16] observation that faith healers are consulted globally for nearly all kinds of ailments including social and psychological issues but mostly refer cases to formal healthcare providers in emergency situations. It was established in this study that that faith users show positive and good attitudes to the healers. Also, it has been noted that key indicators for assessing quality healthcare delivery include users’ attitude, satisfaction and trust. Hence, these indicators play a key role in motivating users to choose one provider over the other [33]. Similarly, Pascoe [34] added that information about users’ satisfaction helps to measure the quality of the service and further serves as a predictor of health-related behaviour of patients. In general, satisfaction with medical care, providers and outcome of treatments are three major areas of measuring patients’ satisfaction regarding the use of healthcare services [35]. Due to this positive and good attitude, most of the study participants chose to visit faith healers frequently more than formal hospitals.

These positive and good attitudes of users toward the healers stemmed from religious and cultural acceptance of faith healing modality. This finding suggests that spirituality, culture and religion have been introduced into the medical circle, implying a growing interest in the possible perceived health benefits connected with having a spiritual belief and/or following a religious belief. Aside the religion, spirituality and culture, good personal relationships and care were also recounted by the participants to account for the positive attitudes toward faith healers. It was inferred from the accounts of the participants that most health care professionals treat their clients with disdain and sheer disrespect. Participants claimed that many health staff do not have the patience to listen to and attend to patients with much care and respect needed.

The decision of patients to choose on provider over the other is premised on effective communication, good interpersonal relationships, carefully listening to patients, being caring, perceived competence and showing compassion [33, 36]. Henceforth, these fearful circumstances psychologically may exacerbate the medical conditions of these patients [37]. These experiences as explained by the participants has dented trust, compelling patients to look for help elsewhere, hence affecting attitudes and care-seeking behaviour of people toward formal health professionals and faith healers respectively. This may also put the unsuspecting patient in much anxieties and worries and resultantly push them into the faith healing utilisation.

These positive attitudes by traditional healing users including faith healing users toward the healers have been widely observed and reported in previous studies [32, 38]. Consistent with the study findings, Leonard [39] argued that faith healing users see the communication between traditional healers and their patients is better compared with formal healthcare providers. Such evidence regarding traditional healers’ service users’ attitude might have accounted for by the familiarity with culture bound syndromes and traditions coupled with their relationships with patients and their families [40].

Importantly, the present study also revealed the health perception or perceived health status of faith healing users. It was found that preponderance of the study participants perceived their health status as good. The study established that users who perceived their health status as poor before their visit perceived their health status as good after the visit to faith healers. This result is in line with other previous study by Levin et al. [27] in America that most users of faith healing services perceived their health status to be good. The perception of participants on their health status after visiting a faith healer reflects how they feel after utilising the prescribed complementary medicines given by the healer. This finding implies that most users often experienced positive improvements in their health status after they had visited and taken the prescriptions by the healer. This also explains why users of faith healing modality perceived the effectiveness of healers’ medicines as good in most times.

Arguably, investigating faith healing modalities’ effectiveness using scientific inquiry is controversial while there is limited evidence of therapeutic effectiveness of faith healing modalities such as prayer [13, 14]. Nevertheless, the study findings proved that most of users have strong believe in the potency of faith healing modality. Most of the study participants perceived faith healing modalities to be very effective for curing, managing, and preventing diseases. Clearly, the participants said that they have effectively been cured from their health problems such as communicable and non-communicable diseases due to their practice of faith healing [4, 31, 41–43]. Specifically, for mental disorders, in the study of resort to faith healing by patients with severe mental illness in Orissa, most people believed in the causative role of black magic bringing mental problems. Most people believed that the person having an abnormal mental state was a victim of external factors, without any problem in body or mind; and considering the nature of this causative factor, faith healing would help rather than medicines [4]. It is, therefore, appears that one culturally prescribed way of dealing with such health problems believed to be caused by another person by action at a distance is resorting to faith healers or the use of counter-power and magic [44].

The reason for selecting faith healers as the first port of contact could be as a result of the perceived effectiveness of faith healing services coupled with the fact that most ailments resulted from spiritual realm and faith healing modality is effective curer of spiritually-motivated diseases. By implication, participants were of the belief that certain diseases have strong spiritual connections. By inference, the utilisation of faith healing is based on perceptions of illness and disease. This perception suggests that diseases must be treated through the exact ways by which they emerged and this is entrenched in faith healing practices. This finding corroborates the discoveries of Osamor and Owumi [45] in urban Nigeria that belief in supernatural causes of illness strongly predicts traditional medicine such as faith healing utilisation.

The study revealed that stigmatization and victimisation were the main challenges of using faith healing services. The study established that patients who utilized faith healing services particularly those who visit spiritualists and oracles more often than not did not receive respect from their neigbours and community members because they were tagged as sinners and non-believers of Jesus Christ. This has implications for the social network, self-esteem and wellbeing of users. This finding is in agreement with previous studies [28, 46, 47]. It, however, contradicts with the findings of Kar [4] that faith healing services should be modified and integrated into the mainstream health care services because patients who received services from faith healing rated it better than treatment from a psychiatric hospital. However, this could be more so when the family, neighbours and the community in which the user finds his or herself have a positive attitude and perception toward faith healing. Thus, a society that generally does not accept faith healing as culturally sanctioned method of healing may stigmatise and victimise faith healing users and vice versa.

Some strengths of the present study need to be remarked. First, to the best of our knowledge, this is the first study of its kind that explores the attitude and health perception of faith healing users in the Kumasi Metropolis, Ghana. The present study proffers an important contribution to address the existing gap in knowledge and also probes Ghana’s health policy framework on a potential regulation of faith healing. However, we also acknowledge a number of limitations. Two important caveats must be added here: Because of the purely qualitative nature of the research that seeks to identify theme that are contextual and therefore cannot be assumed to be independent of the context and individuals, our results should not be regarded as representative to all the general population in Ghana. In line with the interpretive paradigm underpinning this study and the qualitative research methodology adopted, our results cannot be generalized. However, readers can relate the study findings to their settings based on local knowledge. Also, the measures were derived from self-reports of participants of the prior 12-month experiences of faith healing use without recourse to official records or medical diagnosis. Moreover, since this study was conducted among those who were seeking treatment in the faith healing centres, they were a-priori likely to have positive attitude towards the services provided. This therefore expose the findings to potential selection, response and social desirability bias.

### Implications for policy, practice and further research

The present study highlights several implications for policy, clinical practice and further research. First the findings may be relevant to the social policy directions particularly stakeholders involved in the effort to incorporate alternative forms of healing into the conventional health care system in Ghana. The finding is also, particularly relevant to stakeholders in the health sector interested in establishing policy that respects traditional healing systems including faith healing. As policymakers and health professionals debate new health reforms, the understanding of why people consult faith healers for health treatment in Ghana would be an excellent contribution towards the best way forward of intercultural health care implementation. This is because the argument that religion and medicine can partner successfully to instigate wellbeing and mitigate suffering is not a new one as expressed: “Through partnership with faith organisations and the use of health promotion and disease prevention sciences, we can form a mighty alliance to build strong, healthy, and productive communities” [30, 48]. The integration would open doors for formal medical services to have open idea to faith healing.

More importantly, this is the first qualitative study to enrich understanding on attitudes and health perceptions of faith healing users in Ghana. Therefore, the present findings invite further studies involving quantitative and mixed method designs in this arena to provide more nuances into the debate.

## Conclusion

The present study explored the attitudes and health perceptions of faith healing users in the Kumasi Metropolis, Ghana. The study has provided empirical evidence to establish that faith healing users have positive and welcoming attitudes toward faith healers. As a result, faith healers are recognised by users as the 'first port of call' for disease curing, management and prevention of ill-health. Findings also showed that users of faith healing perceived optimal health status largely due to the perceived effectiveness of faith healing modalities. Nevertheless, the study discovered some challenges faced by users, including stigmatisation and victimisation which are mostly associated with the use of faith healing modalities in Ghana. 
