# Correction to: Religion and health: exploration of attitudes and health perceptions of faith healing users in urban Ghana

**DOI:** 10.1186/s12889-018-6326-4

**Published:** 2018-12-20

**Authors:** Prince Peprah, Razak M. Gyasi, Prince Osei-Wusu Adjei, Williams Agyemang-Duah, Emmanuel Mawuli Abalo, Josephine Nii Amon Kotei

**Affiliations:** 1 Department of Geography and Rural Development, Kwame Nkrumah University of Science and Technology, Kumasi, Ghana; 2 African Population and Health Research Center, Manga Close, Off-Kirawa Road, P.O. Box 10787-00100, Nairobi, Kenya

**Correction to: BMC Public Health (2018) 18:1358**

**https://doi.org/10.1186/s12889-018-6277-9**

It was highlighted that the original article [1] contained a typesetting error in the name of Razak M. Gyasi. This was incorrectly captured as Razak M. Gyasi Mohammed in the original article which has since been updated.
